# Phosphorus Release and Adsorption Properties of Polyurethane–Biochar Crosslinked Material as a Filter Additive in Bioretention Systems

**DOI:** 10.3390/polym13020283

**Published:** 2021-01-17

**Authors:** Yike Meng, Yuan Wang, Chuanyue Wang

**Affiliations:** 1 College of Civil and Transportation Engineering, Hohai University, Nanjing 210098, China; 19860001@hhu.edu.cn; 2 College of Water Conservancy and Hydropower Engineering, Hohai University, Nanjing 210098, China

**Keywords:** polyurethane-biochar crosslinked material, modified filter additive, phosphorus release and adsorption, bioretention facilities, stormwater treatment

## Abstract

Bioretention systems are frequently employed in stormwater treatment to reduce phosphorus pollution and prevent eutrophication. To enhance their efficiency, filter additives are required but the currently used traditional materials cannot meet the primary requirements of excellent hydraulic properties as well as outstanding release and adsorption capacities at the same time. In this research, a polyurethane-biochar crosslinked material was produced by mixing the hardwood biochar (HB) with polyurethane to improve the performance of traditional filter additives. Through basic parameter tests, the saturated water content of polyurethane-biochar crosslinked material (PCB) was doubled and the permeability coefficient of PCB increased by two orders of magnitude. Due to the polyurethane, the leaching speed of phosphorus slowed down in the batching experiments and fewer metal cations leached. Moreover, PCB could adsorb 93–206 mg/kg PO_4_^3−^ at a typical PO_4_^3−^ concentration in stormwater runoff, 1.32–1.58 times more than HB, during isothermal adsorption experiments. In the simulating column experiments, weaker hydropower reduced the PO_4_^3−^ leaching quantities of PCB and had a stable removal rate of 93.84% in phosphate treatment. This study demonstrates the potential use of PCB as a filter additive in a bioretention system to achieve hydraulic goals and improve phosphate adsorption capacities.

## 1. Introduction

Phosphorus is the main factor of eutrophication in urban rivers [1] and comes from industry, agriculture and transportation activities [2], being mainly spread by urban stormwater runoff, a kind of non-point pollution of surface water. To manage stormwater runoff, developers typically use bioretention facilities, whose primary goal is to reduce floods by reducing the volume of overland flow during a storm event and reinstating natural stormwater infiltration in the developed area to its pre-developmental capacity [3]. The filtration layer in bioretention facilities takes on the role of purification, which has been proven to be efficient in removing oil [4], heavy metal [5] and pathogenic bacteria indicator species [6] from stormwater runoff. However, the performance of traditional filtration layer is not effective at removing phosphorus [7], mainly due to the leaching of phosphorus from compost (a typical filter additive in the filtration layer) [8].

Attempts have been made to improve phosphorus removal. In recent studies, many natural and artificial materials have been investigated to determine their feasibility as filter additives in the filtration layer of bioretention facilities and they can be generally divided into three types: biological waste materials, mineral materials and biochar. Biological waste materials (e.g., coconut [9], peat [10] and livestock manure [11], etc.) still have high leaching quantities of phosphorus, due to the accumulation of a large number of nitrogen and phosphorus nutrients in the growth process, leading to the limitations of phosphorus removal. Mineral filter additives (e.g., volcanic stone [12], montmorillonite [13] and zeolite [14], etc.) have relatively low removal rates and water retention capacities compared to biological waste materials, in spite of their reduced nutrient-leaching quantities. Biochar, a thermal decomposition product of biomass, is suitable as a filter additive in bioretention facilities due to its cleanness [15] and it can reduce the concentration of both nitrogen and phosphorus in runoff [16]. As a popular soil amendment, biochar can also sequester carbon and retain nutrients [17] and this is significant for additive materials to support the growth of plants in the vegetation layer of bioretention facilities. However, pyrolysis brings brittleness to the pore structure of the biochar, which is destroyed by the hydropower of stormwater during long term operation [18], while the saturated hydraulic conductivity of bioretention facilities is significantly reduced [19]. This does not meet the primary goal of bioretention facilities. If this shortcoming of biochar can be improved, it would be a big step forward for bioretention systems.

Polyurethane materials provide a potentially feasible solution to this problem. On the one hand, the water retention capacity of polyurethane improved under multi-field coupling, due to the broken molecular chain and higher connectivity of the pore structure [20]. When subjected to soil, water and air, the structure of polyurethane showed no significant changes, indicating the good durability of its mechanical properties in the long term [21]. On the other hand, it has been widely recognized that polyurethane foams can be employed as highly efficient adsorbents in removing heavy metals [22], ammonium [23], nitrate [24] and some organic pollutants (e.g., dialkyl phthalates [25], oils and trichloromethane [26], etc.). All these properties meet the requirements of filter additives in bioretention systems: a high hydraulic conductivity to reduce overland stormwater, a high retention volume to minimize peak flow, a good endurance to multi-field coupling effects and a high removal capacity of many contaminants from stormwater. However, due to the limitation of raw material composition, the nutrients needed for vegetation growth cannot be provided by polyurethane alone. Considering the characteristics of biochar, it seems that a combination of polyurethane and biochar may achieve acceptable results.

Additionally, polyurethane, as a coating material, will prolong the nutrient release period of inner fertilizers in agriculture and reduce their leaching quantities [27]. Combined with fertilizers, polyurethane composite material has a high potential to preserve moisture and fertility for the amelioration of desertification [28]. Moreover, this advantage could be applied to biochar in the form of a polyurethane–biochar composite material, helping to release phosphorus more slowly.

Some work has been done regarding polyurethane composites in order to relieve the eutrophication crisis in urban rivers caused by phosphorus—Sasidharan developed silver/silver oxide nanoparticles impregnating polyurethane foam with a 61.24% phosphate and this system was still effective in removing 20.58% of phosphate after 7 cycles of reuse [29]. Nie also conducted a column experiment to purify the septic tank effluent, which was mixed with soil and polyurethane and found that the column had a phosphorus removal rate of 96% [30]. While demonstrating above the positive results in the phosphorus removal of polyurethane composites, these studies are limited and do not estimate the phosphorus leaching quantities of polyurethane composites, nor do they try to apply them to bioretention facilities.

Based on the current research, we assume that, if polyurethane and biochar could be combined together as a composite material with both of their advantages, this composite may have an outstanding hydraulic and environmental performance as a filter additive in bioretention systems. Hence, the present study tried to explore the feasibility of a novel composite material, polyurethane-biochar crosslinked material (PCB), as a filter additive in bioretention systems. We estimated the water retention capacity, phosphorus leaching quantities and adsorption capacity of PCB and aimed to improving the performance of bioretention facilities and avoiding eutrophication. This composite material is a sponge structure in which polyurethane interpenetrates and crosslinks the biochar. Hardwood biochar (HB) was selected as a raw material for the production of PCB because of its low nutrient concentrations [31] and high specific surface area [32] and it was also compared with PCB in this study.

## 2. Materials and Methods

### 2.1. Synthesis of Polyurethane–Biochar Crosslinked Material

- Hardwood biochar (HB) production: the raw material for the synthesis of PCB used in this study is commercially common hardwood biochar, which was produced using pine at a 600 ℃ pyrolysis temperature and was purchased from Jinlian Landscape Engineering Services Co., LTD. (Hangzhou, China).
- Polyurethane-biochar crosslinked material (PCB) preparation: PCB was synthesized with a simple one-shoot method, where the polyol and HB (for modifying polyurethane) were mixed with isocyanate. The polyol source used in this research was glycol and isocyanate was diphenyl-methane-diisocyanate (MDI).
- PCB production: 60 g of glycol, 100 g of deionized water (DW) and 5 g of HB were mixed continuously at 750 rpm and 60 °C for 20 min with a magnetic stirrer (VRera, Nanjing, China). After that, while keeping the same rotating speed and temperature, 250 g of MDI was added dropwise at a constant speed before air bubbles formed. The procedure was continued by pouring the mixture into a 30 × 30 × 10 cm^3^ of mold and transferring it to a vacuum oven (Xidebao, Shanghai, China) at 60 °C for 3 h and then curing it for 24 h.
- Cutting: The cured PCB was cut into granules with a particle size of 1–2mm, considering the practical application and consistent research scale of HB.
- The PCB used in this study was produced with the assistance of Jinlian Company. Scanning electron microscopy (SEM) and energy dispersive spectroscopy (EDS) were conducted on a Hitachi SU3500/S4800 High-Resolution Focused Ion Beam and Scanning Electron Microscope (Hitachi, Tokyo, Japan) working at an accelerating voltage of 10 kV, helping to illustrate the microstructure of PCB and HB.

### 2.2. The Hydraulic Properties and Other Physicochemical Characterizations Tests

The hydraulic property tests include a saturated moisture content test (to evaluate stormwater retention volume) and a permeability coefficient test (to evaluate hydraulic conductivity).

- Saturated moisture content test: The natural bulk densities of the PCB and HB were measured by the cutting ring method (ISO 11272:2017). The samples in the cutting ring were vacuumed by a pump, immersed in deionized water (DW) for 24 h, weighed, dried in an oven at 60 °C for 48 h and weighed again to determine the natural and saturated moisture content (ISO 17892-1:2014).
- Permeability coefficient test: The permeability coefficient of the materials was determined by the constant head method with a Type 70 permeameter (Nanjing Soil Instrument Factory Co., LTD., Nanjing, China) (ISO 17892-11:2019).
- Other physicochemical characterizations influencing the leaching and adsorption capacity of materials were tested:
- The particle size of materials was measured by a sieving method.
- The specific gravity of the materials was measured by the gravity bottle method and the pore ratio of the materials was obtained after conversion with the saturated water content.
- The pH of modifier materials was measured at a material/DW ratio of 1:50 by mass.
- BET surface area was determined by N_2_ (77 K) adsorption on an ASAP 2020 Accelerated Surface Area and Porosimetry System (Micromeritics Instrument, Atlanta, GA, USA) after degassing for 12 h via VacPrep^TM^ 061 (Micromeritics Instrument, Atlanta, GA, USA), a Gas Adsorption Sample Preparation Device.
- The cation exchange capacity (CEC) was determined by a hexamminecobalt (III) chloride solution (ISO 23470:2018).
- TP (total phosphorus content) of materials was measured after strong acid digestion and analyzed by ICP-OES (Thermo Fisher Scientific, Waltham, MA, USA).

### 2.3. Leaching Experiments

The main reason for the unstable efficiency of phosphorus removal in bioretention facilities is the leaching of phosphorus from filter additives, leading to eutrophication in urban rivers. To avoid this pollution and evaluate the phosphorus leaching quantities, polyurethane-biochar crosslinked material (PCB) and hardwood biochar (HB) were continuously rinsed with deionized water (DW) or artificial stormwater (AS) and the release characterizations of phosphorus and metal ions were analyzed. AS was a mixture solution of 120 mg/L CaCl_2_ and 3 mg/L PO_4_-P (Na_2_HPO_4_) at pH 7.0, referring to the recognized makeup of synthetic urban runoff [4]. In order to reduce the influence of other factors, oils, heavy metals and nitrogen were not added to the AS.

Five grams of material, dried at 60 °C for 48 h, was added to a conical flask containing 100 mL of DW or AS (ISO 21268:2019). At 20 ± 2 °C, the material oscillated at a frequency of 150 rpm for 24 h. After settlement for 30 min, supernatants were aspirated into a centrifuge tube and centrifuged at 5000 rpm (Hitachi CR21 Ⅲ, Tokyo, Japan) for 20 min. Another 100 mL of DW or AS was added to the conical flask and the leaching-settling-centrifuging steps were repeated another seven times. The supernatants were filtered with 0.45 μm filters and analyzed for phosphate (PO_4_-P), total phosphorus (TP-P), metal ions (Na^+^, K^+^, Mg^2+^, Ca^2+^) and water conductivity. The San^++^ Continuous Flow Analyzer (Skalar Dutch) was used for the testing of PO_4_-P and TP-P. The samples were mixed with H_8_MoN_2_O_4_, C_8_H_4_K_2_O_12_Sb_2_ and C_6_H_8_O_6_ and a colorimetric analysis was carried out at an 880 nm wavelength for the detection of PO_4_-P with a detection limit of 0.001 mg/L (ISO 15681:2018). The method for the detection of TP-P was the same as PO_4_-P, only needing a pretreatment (oxidized by K_2_S_2_O_8_ solution and digested by UV) before mixing. Metal ions were tested by using a NexION300X inductively coupled plasma mass spectrometer (PerkinElmer, Waltham, MA, USA) with a detection limit of 0.001 mg/L. Conical bottles containing only DW or AS without other materials were used as the control groups. We repeated two sets of tests for each material. After leaching experiments, the DW-rinsed materials were denoted as PCB-DW and HB-DW respectively and then put into a desiccator for later tests.

### 2.4. Phosphate Adsorption Experiments

As a filter additive in stormwater runoff treatment, the material needs to have a certain phosphate adsorption capacity. In order to evaluate the phosphate adsorption capacity of the materials, adsorption experiments were conducted using PCB-DW and HB-DW with different concentrations of phosphate. Standard solutions of 100 mg/L Na_2_HPO_4_ were diluted to 0, 0.5, 1, 2, 5, 7 and 10 mg/L with DW and AS, respectively and AS only contained 120 mg/L CaCl_2_. We placed 0.2 g of PCB-DW and HB-DW into 50 mL conical flasks, added 10 mL of the above solution and oscillated the flasks for 24 h at 150 rpm at 20 ± 2 ℃. The extraction method of the supernatants was the same as the leaching test and the concentrations of phosphate in the supernatants were measured. The detection method was the same as above. The experiment was repeated in 2 groups for each material. Additional conical flasks with phosphate solutions but no PCB-DW or HB-DW were used as control groups.

In order to explore the adsorption properties and capacity of phosphate, Langmuir and Freundlich models were used to fit the adsorption equilibrium quantities of phosphate after 24 h. The calculation formula of the equilibrium adsorption quantity *q_e_* (mg/kg) for phosphate at 24 h is [33]:

(1) $$q_{e}^{}=\frac{(C_{0}-C_{e})V}{W},$$

where, *C*_0_ and *C_e_* are the concentrations (mg/L) of PO_4_^3−^ in the solution before and after the adsorption test; *V* is the solution volume (L); *W* is the material mass (kg).

The Freundlich model was used to fit the isothermal adsorption results [23]:

(2) $$q_{e}=K_{F}Ce^{1/n},$$

where, *K_F_* is the volume-affinity parameter (L/mg) of the Freundlich model, which can be regarded as the adsorption capacity at unit a concentration of *C_e_*; *n* is the Freundlich characteristic constant, the value of *1/n* is generally between 0 and 1 and its value represents the influence of the concentration on the adsorption capacity. The smaller *l/n* is, the better the adsorption property is. When *1/n* is between 0.1 and 0.5, it is easy to absorb; it is difficult to adsorb when *1/n* is more than 2.

The Langmuir model of single molecular layer physical adsorption was also used to fit the isothermal adsorption results [23]:

(3) $$q_{e}=q_{\max}\frac{K_{L}C_{e}}{1+K_{L}C_{e}}$$

where *q_max_* is the maximum adsorption capacity (mg/kg); *K_L_* is the affinitive parameter of the Langmuir model (L/mg), which is the equilibrium constant of adsorption, also known as the adsorption coefficient. The higher the value of *K_L_* is, the stronger the adsorption capacity is.

The dimensionless coefficient *R_L_* is used to determine whether adsorption easily occurs [34]:

(4) $$R_{L}=\frac{1}{1+K_{L}C_{0}},$$

when 0 < *R_L_* < 1, adsorption easily occurs; when *R_L_*>1, adsorption does not easily occur; when *R_L_* = 0, the adsorption process is reversible. When *R_L_* = 1, adsorption is linear.

### 2.5. Column Experiments

In order to simulate the performance of the filter additives under an actual working situation, column experiments were carried out in the laboratory. The river sand (washed by DW and dried) and filter additives (unwashed) were mixed evenly according to a mass ratio of 10/1. In 3 PVC columns (30 cm in height, 6 cm in diameter and 3 mm holes of sieve tray at the bottom), filter papers were placed at the bottom and evenly mixed geomedia filled the columns, denoted as PCB-Column, HB-Column and Sand-Column. Sand-Column was filled only with river sand as a control group. For each 2 cm of mixed filling, a wooden hammer was employed to drop 10 times from 5 cm above the filling until it was filled to 24 cm. Washed and dried gravels were placed on top of the mixture filling to prevent current scour. After filling, peristaltic pumps (BT01-100) were used at the top of the columns to pump DW for 3 h at a speed of 15 mL/min. The inflow velocity was calculated according to the rainfall intensity formula in China. In this study, rainfall occurred once a year and lasted for 3 h, the catchment ratio was 15 and the infiltration flow per minute was calculated as 15 mL. In total, 50 mL of effluent was collected every 20 min by the effluent tubing at the bottom in order to detect the contents of PO_4_-P. The detection methods were the same as above. AS was pumped at the same rate for 3 h after the DW was pumped in for 3 h. AS included 3 mg/L PO_4_-P. Effluent was collected and measured as above.

## 3. Results and Discussion

### 3.1. Polymerization Process and Microstructure

**Figure 1:**
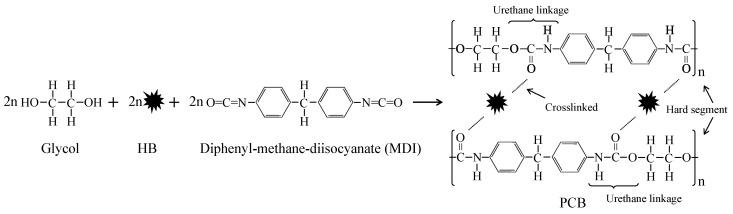
The reaction of polyurethane-biochar crosslinked material (PCB) via the one–shoot method with the addition of HB.

The Polyurethane-biochar crosslinked material (PCB) was obtained via a one-shoot method. The reaction of PCB and the interaction with the addition of HB are shown in Figure 1. Glycol contained a hydroxyl group that could reacted with an isocyanate group from MDI to obtain a urethane linkage and a monomeric unit was formed by the two constitutional units. Furthermore, the polymer chain was linked through urethane linkages between monomeric units. HB was crosslinked between two monomeric units [22].

**Figure 2:**
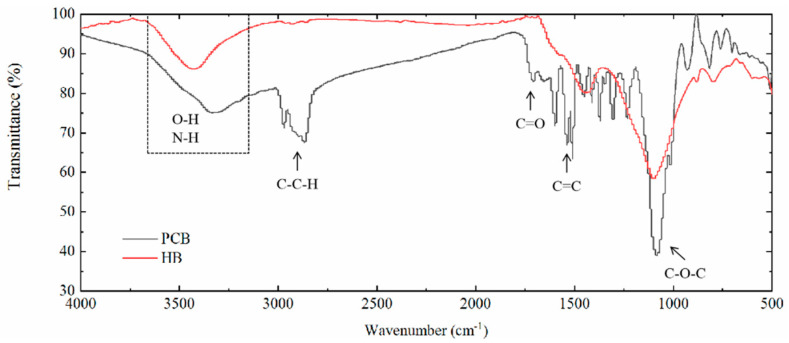
Fourier transform infrared (FTIR) spectra of PCB and hardwood biochar (HB).

The properties of PCB depend on various factors (chain rigidity, cross-linking degree, intermolecular bonds, etc.) and can be changed in a wide range by the proper selection of raw materials [35]. Considering the application of PCB in bioretention facilities, durability, resilience, porousness and hydrophilia were demanded by the multi-field (water-soil-air) coupling effects. Glycol was chosen as the main polyol source that would reduce the length of carbon chain and improve the hard segment content. The hard segment can improve the initial modulus and tensile strengths of polyurethane materials [36] and crosslinked polymerization would make up for the brittleness of HB to make it resilient against weathering. HB, as an inner material, was blocked in the network of polyurethane by the interaction between carbonyl groups and HB. The blocking way was referred from the FTIR analysis (Figure 2): The interaction between the HB and polyurethane was observed by the shifted wavenumber of carbonyl groups at around 1600 cm^−1^. As a carbon-rich material, HB possessed abundant hydroxyl groups [37] and was more likely to have this interaction [38].

**Figure 3:**
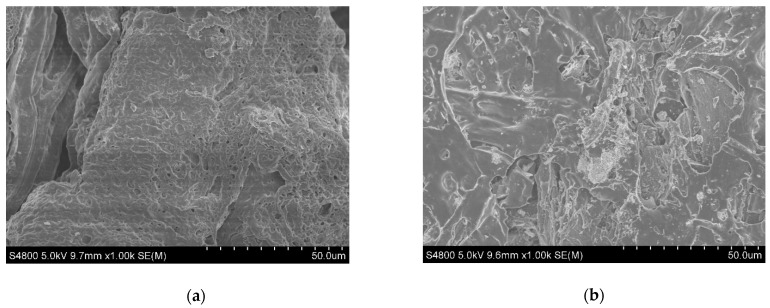
The scanning electron microscopy (SEM) images of (**a**) PCB and (**b**) HB.

The polyol source also influenced the microstructure of polyurethane composites [39]. Small molecular weight polyol has a better smoothness and porousness. As shown in Figure 3, the concave-convex surfaces and throats of PCB can be clearly seen, yet HB has a flat shape and few holes and bumps. This difference in structure may account for the improvements in the hydraulic properties of PCB and HB.

### 3.2. Hydraulic Properties of Modifiers

Excellent hydraulic performance, including high water retention capacity and permeability, are fundamental criteria for choosing filter additives in bioretention systems, which are also shortcomings of biochar at present [19], when compared to biological waste and mineral materials. Basic experiments were conducted to inspect the improvement of PCB and the results are illustrated in Table 1. After being modified by polyurethane, the material became lighter, with a bulk density of 0.165 g/cm^3^, compared to its former bulk density of 0.378 g/cm^3^. Such lightweight polyurethane gave PCB a sizable performance boost in water retention capacity [40], whose saturated water content was improved from 195.65% to 383.5%. If added to the filter layer with the same mass ratio (4% of additive materials in traditional bioretention facilities [41]), PCB can reduce 42–63 mm of stormwater within the unit area according to the following water absorption formula:

(5) Wwater=ρfilterlayer×h×n×ωsat,

where *W_water_* is the stormwater retention volume of the filter additive within unit area; *ρ_filterlayer_* is the density of the filter layer in bioretention facilities, ranging from 0.8 to 1.2 g/cm^3^; *h* is the filling height, usually calculated as 70 cm; *n* is the mass ratio of the filter additive; *ω_sat_* is the saturated moisture content of the filter additive.

This improvement is of great significance and will achieve a high storage volume in order to reduce peak flow and enhance the removal of many contaminants from stormwater [42]. The Permeability of HB was also improved by the polyurethane polymerization, the infiltration coefficient of which changed from 6.57 × 10^−4^ to 8.56 × 10^−2^, with an improvement of more than two orders of magnitude. Apart from the bigger particle size, as an influencing factor on permeability, the internal throats formed in the foaming process also took effect, where stormwater flowed inside the network through the throats, increasing the number of available flowing paths [43].

### 3.3. Phosphorus Leaching

**Figure 4:**
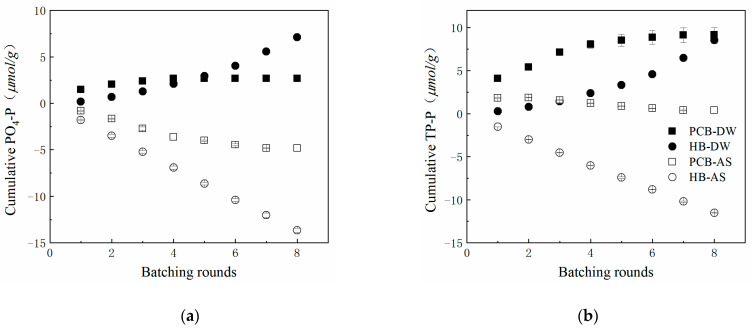
Cumulative phosphorus compounds leached from PCB and HB in DW or AS. (**a**) PO_4_-P; (**b**) TP-P.

Before application in bioretention facilities, the quantities of phosphorus that could leach from additive materials should be estimated to prevent potential eutrophication. PO_4_-P and TP-P were detected in the successive leaching solution and the results are shown in Table 2 and Figure 4.

In general, PCB released more than HB in the first DW batching round: 1.47 μmol/g of PO_4_-P and 4.09 μmol/g of TP-P for PCB, while 0.19 μmol/g of PO_4_-P and 0.27 μmol/g were released for HB. After the first batch, the leaching quantities of phosphorus were in decline in each round and PCB released 2.68 μmol/g of PO_4_-P and 9.16 μmol/g of TP-P in total, while these values were 7.11 and 8.55 for HB, respectively. Compared to the compost (around 82 μmol/g of PO_4_-P in 6 rounds of leaching) [11] and poultry litter biochar (82.6–146.1 μmol/g of PO_4_-P in 10-days leaching) [15] in other studies, the phosphorus leaching quantities of PCB and HB were relatively low.

The inhibition effects of crosslinked polyurethane on the phosphorus leaching of HB can be observed from the different leaching tendencies of the two materials: The leaching quantities of HB increased as the number of rounds increased and it continued to release more and more PO_4_-P and TP-P at a nearly constant rate. The leaching tendency of HB was in agreement with former research on biochar leaching properties [44] and it can be predicted that additional phosphorus would be released with further batching. However, after being crosslinked and interpenetrated by the polyurethane, the release of phosphorus from the internal biochar was prevented, as previously reported [27]. PCB’s first round of phosphorus leaching accounted for 44.65–54.85% of the total released quantities and the released quantities were only in the range of 2.67–3.16% for HB. The resilient and smooth network of polyurethane could resist scour caused by water and prevent itself from weathering. Polymerization made the HB and polyurethane blend seamlessly, which avoided phosphorus on the surface of HB being washed away by waterpower. The reason for PCB releasing more TP-P than HB could be that the surface of PCB was brushed with organophosphorus flame retardants to meet the storage and transportation conditions [45].

Since the AS contained 3 mg/L of PO_4_-P, it was subtracted when calculating the cumulative phosphorus compounds leached from PCB and HB in AS, so the data present negative values. Our assumption from the negative values was that PCB and HB had a certain adsorption capacity to the 3 mg/L of PO_4_-P in AS. HB had a better treatment effect on phosphorus, whose adsorption capacity was unimpeded by batching rounds. It is likely that the alkalinity of HB (Table 1) brought this benefit, which could provide an alkaline condition to form hydroxyapatite precipitation with Ca^2+^ and PO_4_^3-^ in stormwater runoff [46]. Meanwhile, PCB had a relatively poor treatment performance on phosphorus due to its acidity in the water. It is believed that PCB also had a slight effect on phosphorus adsorption, reducing the leaching quantities of PO_4_-P and TP-P in AS. The mechanism of PCB phosphorus adsorption could be ion exchange or physical adsorption but this is inconclusive.

### 3.4. Leaching of Other Ions

**Figure 5:**
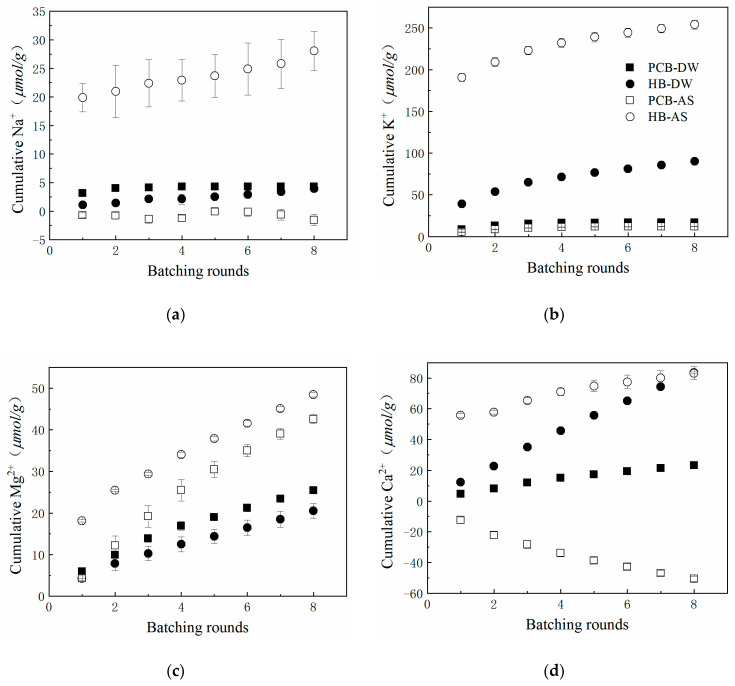
Cumulative low valence metal cations leached from PCB and HB in DW or AS. (**a**) Na^+^; (**b**) K^+^; (**c**) Mg^2+^; (**d**) Ca^2+^

The main commercial processes for removing phosphorus from wastewater effluents are still chemical precipitation with metal ions [46]. The leaching pattern of metal ions could help to elucidate the mechanisms of the removal of phosphorus by PCB and HB. The leaching of low-valence metal ions (Na^+^, K^+^, Mg^2+^, Ca^2+^) as a function of batching rounds is shown in Figure 5 and summarized in Table 3. Generally, PCB leached fewer or nearly equal numbers of metal ions than HB in DW. AS prompted the metal ion-leaching quantities of HB but had no obvious impact on PCB.

Na^+^ was leached at a very low level in DW. From the energy dispersive spectroscopy results (Table 4) of PCB and HB, there was no Na^+^ on the analyzed surface of PCB but a small quantity on the surface of HB (0.62%). Hence, the low quantities of Na^+^ in DW were reasonable. PCB and HB had similar cumulative release amounts but their release rates and patterns were not consistent: PCB released 3.13 μmol/g Na^+^ in the first round, accounting for 73.05% of total release quantities and released less during batching rounds. While HB only released 1.10 μmol/g Na^+^ in the first round, after that it leached at an almost constant rate. Because of the valence being the same, the pattern of K^+^ leaching was similar to Na^+^: the cumulative Na^+^/K^+^ leaching quantities increased logarithmically, which was consistent with previous research on biochar leaching [47]. AS motivates more metal ions to leach out from HB in the first round but keeps an approximate leaching rate afterwards, as in DW. The leaching quantities of PCB were significantly lower than HB, probably because the K^+^ on the surface of HB had been released in the glycol–DW mixing process, most of which was cured in the inner structure of PCB during polymerization process.

The Mg^2+^ was released from PCB and HB linearly and a nearly equal amount of Mg^2+^ was released in each batching round. EDS results (Table 4) showed that there were 0.74% Mg elements on the surface of HB before leaching which could not be detected after leaching. This indicated that Mg^2+^ adhered to the surface of HB in the form of mineral ions and was washed away. The Mg^2+^ content on the surface of PCB dropped slightly after 8 rounds batching and this suggested that it mostly existed as compounds or was blocked in the polyurethane network. In AS, total Mg^2+^-releasing quantities and speed increased compared with those of DW: the first-round leaching quantity of PCB in AS was close to that of DW and the release rate of Mg^2+^ in AS increased to 1.22–1.67 times that of in DW in the later leaching process. This increase indicated that the removal of phosphorus was not due to the magnesium–phosphorus precipitation.

The Ca^2+^ leaching rates of PCB and HB seems to be constants whether in DW or AS and are unaffected by the batching rounds. HB leached significantly more Ca^2+^ than PCB, since EDS results showed that HB had a higher Ca^2+^ content than PCB on the surface. Interestingly, HB leached the same Ca^2+^ quantities in AS as in DW, yet with a higher concentration in the first round. The equality of the leaching quantities proved the previous assumption that the reduction of PO_4_-P was caused by formation of hydroxyapatite precipitation with Ca^2+^ and PO_4_-P in stormwater runoff. On the contrary, the cumulative Ca^2+^-releasing quantities of PCB were negative and underwent a steady decline, consistent with the tendency of phosphorus leaching but unbalanced in their quantities. This made the mechanism of PCB adsorption to decrease phosphorus concentration uncertain. This could be partly attributed to the calcium–phosphorus precipitation when considering the negative value of Ca^2+^ releasing quantities but other phosphorus-removing approaches coexisted.

Overall, the metal cations of HB, existing as salts on its surface, were easy to washed away, especially for K^+^ and Ca^2+^ and this was proved by the SEM and EDS results. After the modification of crosslinked polyurethane, the leaching quantities of metal cations were significantly reduced. Through the correspondence of the leaching quantities between phosphorus and metal ions, it is clear that the mechanism of phosphorus removal by HB is calcium–phosphorus precipitation. PCB has several phosphorus removing approaches, including metal salts precipitation, which requires further research.

### 3.5. Phosphate Adsorption

**Figure 6:**
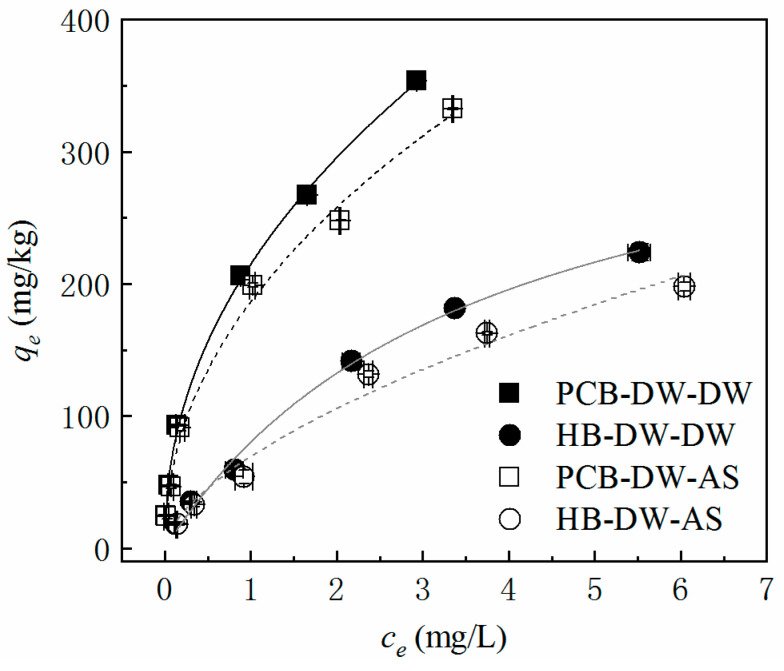
Adsorption isotherms of PCB-DW and HB-DW in DW or AS.

Isothermal adsorption experiments can help to estimate the adsorption capacity and properties of additive materials in a bioretention system. The phosphate adsorption results of PCB-DW and HB-DW in DW and AS are shown in Figure 6. PCB-DW had a compelling advantage in phosphate adsorption to contrast to HB-DW: PCB-DW had a stronger equilibrium adsorption capacity, which was 1.32–1.58 times of that of HB-DW. The adsorption rates of PCB-DW were 70–98% under at different concentrations, while HB-DW could adsorb 44–74% of phosphate. PCB-DW and HB-DW adsorbed more in DW than in AS over the tested concentration range but with minimal growth. At a typical phosphate concentration range of 2–5 mg/L in stormwater runoff, the equilibrium adsorption was 93–206 mg/kg for PCB and 60–142 mg/kg for HB.

As confirmed by previous research, phosphate was bound to the biochar not only by electrostatic adsorption but also by covalent bonds, forming highly valent cationic-phosphate crystals, including magnesium [48], iron, alum or calcium [46]. There are many factors and complex evolvement courses for PO_4_-P adsorption by polyurethane: the main mechanism of phosphate removal is adsorption, which occurs as a result of electrostatic attraction between two oppositely charged ions, where pH plays an important role, preferring to remain around 7 [29]. This explained why PCB (pH = 6.62) showed a poorer adsorption capacity in the leaching experiments but PCB-DW (pH = 6.98) did better in the isothermal adsorption experiments. Adsorption in AS was inferior to that in DW, which indicated that additional Ca^2+^ in AS could not promote the progress of precipitation, instead weakening it and the bivalent and multivalent cations leaching from themselves were adequate for removing phosphorus. In this study, the superiority of PCB-DW was the multiple factors, including the weak acidic conditions with a pH around 7, an abundant supply of bivalent and multivalent cations and a relatively high BET (Table 1). A thorough, quantitative analysis of the factors of phosphate adsorption is still required, however.

The results of the isothermal adsorption of PCB-DW and HB-DW were also fitted to two adsorption models, as shown in Table 5. The Freundlich model fitted the PO_4_-P adsorption data of the PCB-DW better, with *R*^2^ > 0.99, while the Langmuir model was better for HB-DW. Ahmed also found that the Freundlich model had the best fit for the adsorption of nutrients onto polyurethane materials [23]. With the inverse of the characteristic constants (*1/n*) < 1 in Freundlich models and the Langmuir model coefficient *R_L_* being between 0 and 1, we can draw the conclusion that the adsorption of PO_4_-P occurred easily for both PCB-DW and HB-DW. The adsorption of PO_4_-P by PCB-DW and HB-DW was nonlinear. With the increase in the PO_4_-P concentration in the solution, its adsorption capacity gradually becomes saturated, which was also confirmed by the bending of the fitting curves in Figure 6. *K_F_* (the Freundlich model’s volumetric-affinity parameter), to some extent, proved that, compared to HB-DW, PCB-DW had a better adsorption affinity for phosphate. The *q_max_* in the Langmuir model reflected the potential maximum adsorption capacity of the materials and PCB-DW had higher *q_max_* than HB-DW no matter in DW or AS. Therefore, PCB-DW can be used as an additive with high adsorption performance in stormwater treatment.

### 3.6. Stormwater Infiltration Experiments

**Figure 7:**
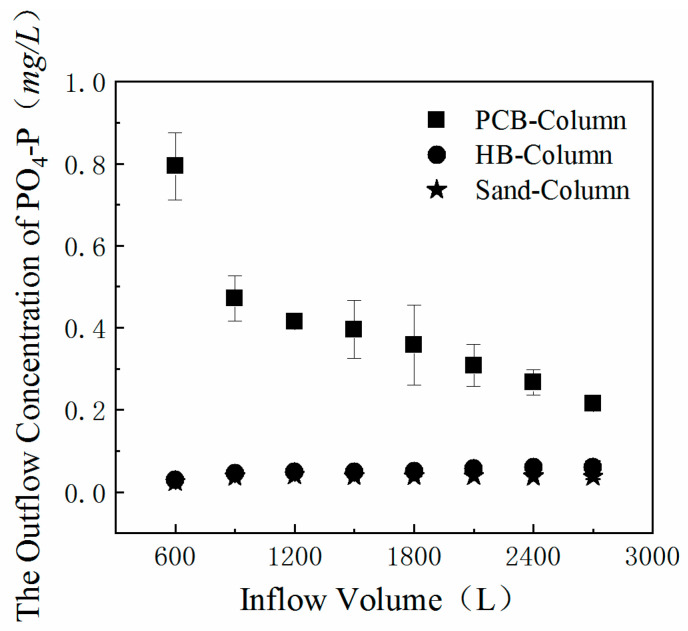
Concentration of PO_4_^3−^ in outflows from the three columns in the first simulated rainfall event.

In order to explore the actual operation effects after PCB and HB are added into bioretention facilities as additives, column tests were conducted, simulating the rainwater infiltration process and evaluating the suitability and feasibility of the materials. Two simulated rainfall events were carried out, each of which lasted for 3 h. DW was pumped into three columns at a rate of 15 mL/min in the first event. The PO_4_-P concentrations of outflows were detected every 20 min and the variations in the outflow concentrations are shown in Figure 7. The outflows from the PCB-Column had higher PO_4_-P concentrations compared with the HB-Column. With the increase in DW inflow volume, the effluent concentration decreases linearly, nearly to 0. Traces of PO_4_-P were detected in the outflow of the HB-Column, while the content rose slightly during the infiltration process. The tendency of the concentration to grow in the HB-Column outflow was in accordance with the leaching patterns in the batching experiments.

The estimated and detected values of the PO_4_-P concentration and total release quantities from PCB-Column and HB-Column are listed in Table 6. The estimate was based on the assumption that the PCB (626.73 g) and HB (834.94 g) in the soil columns would release the same quantities of phosphorus as in the leaching tests. Comparisons in Table 6 illustrated the wide gaps between the estimated and the detected values. These gaps were caused by the differences in the contact ways and the hydrophilicity of materials, which could also be predictable. In column infiltration experiments, the DW inflow had a shorter contact time and a smaller contact surface with the additive PCB and HB and reduced the leaching quantities. Gupta drew a similar conclusion through the observation of heavy metal batch leaching experiments and column experiments [49]. HB was pyrolyzed at 600 ℃ and oxygenated functional groups on HB’s surface made it possess a lower hydrophilicity [50]. The percentage of polyol used as a raw material in polyurethanes is positively associated with hydrophilicity [51] and the molar ratio of urethane linkage:polyol in PCB was 1:1. Combined with the water retention capacity of PCB, we could infer that PCB had a higher hydrophilicity. Additionally, the volume of PCB was double that of HB under same ratio in column experiments and PCB had more contact time and space in the stormwater. Hence, it made sense that there were more PO_4_-P leaching quantities in the PCB-Column than in the HB-Column.

**Figure 8:**
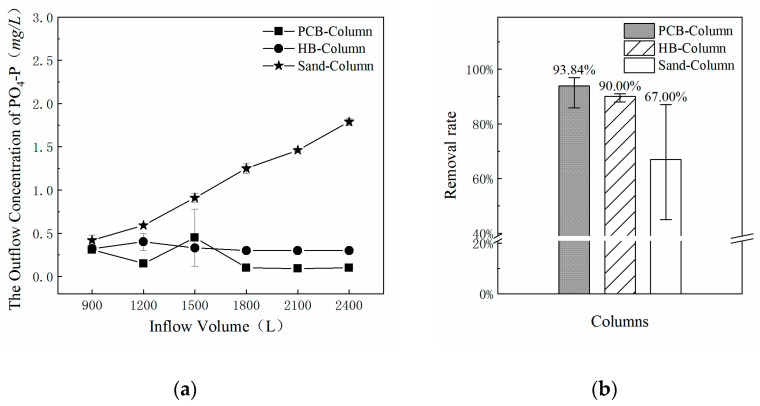
Concentration and mean removal rates of PO_4_^3−^ in outflows from the three columns in the second simulated rainfall event. (**a**) The outflow concentrations of PO_4_^3−^ from the three columns; (**b**) the mean removal rates of PO_4_-P from the three columns.

DW was replaced by AS in the second simulated rainfall event, while the other conditions remained. Considering the influence of the first rainfall event, the PO_4_-P concentration of the outflow was detected after the infiltration for 1 h and the results are shown in Figure 8. The PO_4_-P concentration of the outflow from the PCB-Column and HB-Column remained stable at lower levels, while it increased sharply from the Sand-Column during the AS infiltration process. MeanPO_4_-P removal rates for the three columns during the AS flushing are also shown in Figure 8. Compared with the control group (Sand-Column), the experimental groups (PCB-Column and HB-Column) had a significantly higher PO_4_-P filtration capacity, with removal rates of 93.84% and 90.00%, respectively. This confirmed the feasibility and superiority of PCB as a filter additive in bioretention systems for removing PO_4_-P in stormwater treatments.

In addition, it should be noted that, in the effluent concentration detection, the concentration of PO_4_-P was kept at a low and stable state with a downward trend, which confirmed the influence of hydrophilicity on the adsorption effect of filter additives. With the infiltration and scouring of water inflow, oxygenated functional groups carried on the surface of HB were gradually washed away, so its hydrophilicity was improved to some extent. For polyurethane, its surface roughness changed during the infiltration process, which affected its adsorption capacity [52]. After the increase in hydrophilicity and surface roughness, the contact paths and time between stormwater inflow and filter materials increased, leading to the enhancement of adsorption. Although the improvement of hydrophilicity and surface roughness led to the enhancement of the adsorption capacity, the adsorption capacity tended to be saturated as the adsorption process continued. Therefore, the concentration of the outflow did not decrease significantly and it maintained a relatively stable trend in later stages.

## 4. Conclusions

Polyurethane-biochar crosslinked material (PCB) has been successfully manufactured using polyurethane and hardwood biochar (HB) in order to improve the hydraulic performance of bioretention facilities. This improvement was confirmed through a characteristic analysis of PCB using FTIR spectra, SEM images and hydraulic parameter tests. Biochar was crosslinked through urethane linkages in the polyurethane network. Saturated water content was doubled due to the hydrophilia and porousness of polyurethane. The internal throats, confirmed by the SEM images, increased the permeability coefficient of the filter additive by two orders of magnitude.

From the perspective of phosphorus release and adsorption, PCB is a feasible filter additive in bioretention facilities for stormwater treatment. The network of polyurethane restrained the release of phosphorus from interpenetrated HB with a decreasing cumulative rate of phosphorus leaching and reduced the metal cation leaching quantities compared with HB. The superiority of the adsorption capacity of PCB should be emphasized: for the typical phosphate concentration of stormwater runoff, the equilibrium adsorption quantity of PCB is 93–206 mg/kg for phosphate, which is a result of various factors, including its suitable pH, cation supply and porousness. PCB has a high (93.84%) and stable phosphate removal rate in column experiments, owing to the hydrophilia and porousness of polyurethane.

Overall, the present study offers a feasible filter additive with modified hydraulic properties and environmentally friendly advantages for bioretention facilities to use in stormwater treatment. Changes in the properties via the adjustment of the formula and ratio in PCB preparation lead to a variation in pore size and the functional group should be examined in further research. Meanwhile, the effect of PCB on the removal of other pollutants in stormwater should be investigated in the future.

## Figures and Tables

**Table 1:** Physicochemical properties of PCB and HB.

Media Material	*ρ*^1^ (g/cm^3^)	Particle Size (mm)	*e* ^2^	*ω_sat_*^3^ (%)	*K*^4^ (cm/s)	pH	BET (m^2^/g)	CEC (cmol/kg)	TP ^5^ (g/kg)
PCB	0.165	1–2	3.20	383.50%	8.56 × 10^−2^	6.62	83.14	37.5	1.19
HB	0.378	<0.5	3.88	195.65%	6.57 × 10^−4^	8.80	118.45	7.4	3.80

^1^*ρ* is natural bulk density. ^2^ *e* is pore ratio. ^3^ *ω_sat_* is saturated moisture content. ^4^ *K* is permeability coefficient. ^5^ TP is total phosphorus content.

**Table 2:** Phosphorus leaching quantities of PCB and HB in deionized water (DW) or artificial stormwater (AS).

Media Material	PO_4_-P			TP-P			
	8 rounds (μmol/g)	1 round (μmol/g)	1 round/ 8-rounds	8 rounds (μmol/g)	8 rounds/ Total	1 round (μmol/g)	1 round/ 8 rounds
PCB-DW	2.68	1.47	54.85%	9.16	23.86%	4.09	44.65%
HB-DW	7.11	0.19	2.67%	8.55	6.98%	0.27	3.16%
PCB-AS	−4.82 ^1^	−0.82 ^1^	-	0.38	-	1.81	-
HB-AS	−13.67 ^1^	−1.79 ^1^	-	−11.51 ^1^	-	−1.53 ^1^	-

^1^ The negative value came from the original concentration of AS solution (3 mg/L PO4-P), which was subtracted in a calculation and indicated a decrease in AS.

**Table 3:** Cations leaching quantities of PCB and HB in DW or AS.

Media Material	Na^+^			K^+^			Mg^2+^			Ca^2+^		
	8 rounds (μmol/g)	1 round (μmol/g)	1 round/ 8 rounds	8 rounds (μmol/g)	1 round (μmol/g)	1 round/ 8 rounds	8 rounds (μmol/g)	1 round (μmol/g)	1 round/ 8-rounds	8 rounds (μmol/g)	1 round (μmol/g)	1 round/ 8 rounds
PCB-DW	4.28	3.13	73.05%	16.78	8.67	51.69%	25.48	5.92	23.23%	23.27	4.71	20.22%
HB-DW	3.91	1.10	28.04%	90.18	39.16	43.42%	20.55	4.29	20.90%	83.56	12.39	14.83%
PCB-AS	−1.53	−0.72	-	12.07	5.39	44.66%	42.59	4.33	10.17%	−50.65	−12.45	-
HB-AS	28.04	19.87	-	254.03	190.68	75.06%	48.45	18.15	37.46%	83.16	55.63	-

**Table 4:** Energy dispersive spectroscopy (EDS) contents of original and DW-leached materials.

Elements	PCB	PCB-DW	HB	HB-DW
	*W_t_*%			
C	58.74	55.89	38.16	31.93
O	35.18	37.64	32.04	38.17
Na	-	-	0.62	0.27
Mg	0.83	0.73	0.74	-
Al	0.83	0.31	1.56	0.37
Si	1.26	0.24	17.61	25.96
K	0.64	-	3.16	0.86
Ca	2.52	5.19	6.11	2.44
Total	100			

**Table 5:** Parameters for Freundlich and Langmuir isotherms of phosphate adsorption on PCB-DW and HB-DW.

Material	Freundlich			Langmuir			
	*K_F_* (L/mg)	*1/n*	*R* ^2^	*q_max_* (mg/kg)	*K_L_* (L/mg)	*R* ^2^	*R_L_*
PCB-DW-DW	214.978	0.460	0.998	417.833	1.3156	0.959	0.071–0.603
HB-DW-DW	80.500	0.623	0.983	374.176	0.274	0.994	0.267–0.880
PCB-DW-AS	186.782	0.467	0.990	379.160	0.510	0.960	0.164–0.797
HB-DW-AS	69.599	0.606	0.977	319.12	0.276	0.990	0.266–0.879

**Table 6:** Comparison of predicted and detected concentrations and total release of PO_4_^3-^ in column experiments.

Columns	Concentration of the First 50 mL Effluent (mg/L)		Total Leaching Quantities (mg)	
	Predicted	Detected	Predicted	Detected
PCB-Column	47.60	0.80	52.07	1.22
HB-Column	8.20	0.03	184.03	0.13

## Data Availability

The data presented in this study are available in this article.
